# Overshadowing and salience attribution in relation to cannabis use

**DOI:** 10.1016/j.scog.2024.100315

**Published:** 2024-05-10

**Authors:** Christopher Dawes, Samuel Joy McGreal, Shivika Marwaha, Jose Prados, Antoine Reheis, Alin Dumitrescu, John L. Waddington, Paula M. Moran, Colm O'Tuathaigh

**Affiliations:** a School of Psychology, University Park, Nottingham NG7 2RD, UK; b School of Medicine, University College Cork, Cork, Ireland; c School of Psychology, University of Derby, Derby DE22 1GB, UK; d Psychological Sciences Research Institute, Université Catholique de Louvain, Louvain-la-Neuve, Belgium; e St. Stephen's Psychiatric Hospital, Sarsfield Court, Glanmire, Cork, Ireland; f School of Pharmacy and Biomolecular Sciences, RCSI University of Medicine and Health Sciences, St Stephen's Green, Dublin 2, Ireland; g Medical Education Unit, School of Medicine, University College Cork, Cork, Ireland

**Keywords:** Cannabis, Overshadowing, Aberrant salience

## Abstract

Aberrant attentional salience has been implicated in the cannabis-psychosis association. Here, history and frequency of cannabis use were examined against changes in overshadowing (OS), a cue competition paradigm that involves salience processing. Additionally, we examined the association between OS and alternative measures of aberrant salience, as well as schizotypy, in a non-clinical adult sample.

280 participants completed an online geometry learning-based OS task, while a subset (*N* = 149) also completed the Salience Attribution Task (SAT) measure of aberrant salience. All completed the Schizotypal Personality Questionnaire (SPQ), Aberrant Salience Inventory (ASI), and the modified Cannabis Experience Questionnaire (CEQmv). Differences across OS and SAT performance stages and between cannabis use groups were assessed using mixed ANOVAs. Multiple regression and correlational analyses assessed the relationships between OS and SAT task metrics and SPQ and ASI subscale scores.

Current cannabis users had significantly lower OS scores during the testing phase relative to those who do not use cannabis, at medium effect sizes. Schizotypy or ASI scores did not mediate this relationship. In the SAT, current cannabis users presented significantly higher implicit aberrant salience relative to non-users. Scores in the first training phase of the OS task significantly predicted higher explicit aberrant and adaptive salience scores in the SAT.

These data indicate an association between regular cannabis use and abnormalities in cue competition effects in a healthy adult sample. Comparisons of OS and SAT cast new light on putative overlapping mechanisms underlying performance across different measures of salience.

## 1 Introduction

Prevalence of cannabis use during the past decade has increased by 27 % in European adults, from 3.1 % to 3.9 % (Manthey et al., 2021). Worldwide, the increasing trend towards legalisation and commercial sale of recreational cannabis has brought increased attention to its association with risk for psychosis (Murray and Hall, 2020). A study in Denmark found that almost half of harm associated with cannabis use across the population was seen in patients with schizophrenia (Weye et al., 2021). A large number of studies have found that cannabis use is associated with psychosis. A meta-analysis of meta-analytic studies and systematic reviews concluded that psychotic illness arises more frequently in cannabis users compared to non-users, cannabis use is associated with a dose-dependent risk of developing psychotic illness, and frequent cannabis users have an earlier onset of psychotic illness compared to non-users (Hasan, 2019). Frequent cannabis use is associated with increased severity of psychotic symptoms, with factors including frequency of use, age at first use and potency of cannabis moderating that risk (Colizzi and Murray, 2018; Di Forti et al., 2019; Lee Pow et al., 2023). Cannabis use was also associated with increased relapse rates, more hospitalizations and pronounced positive symptoms in psychotic patients.

Despite the well-established link between cannabis use and greater risk for developing a psychotic disorder, the psychological mechanisms underlying this association remains poorly understood. One potential mechanism is via abnormal processing of salience information. Bhattacharyya et al. (2012) showed that THC administration in healthy volunteers induces psychotic symptoms and disrupts visual oddball detection, a task that addresses attentional salience. There is also good evidence from fMRI studies that cannabis administration and even simple visual exposure to cannabis cues is associated with patterns of activation and deactivation in brain regions consistent with salience processing such as the caudate and prefrontal cortex (Bhattacharyya et al., 2012; Charboneau et al., 2013). In positron emission tomography (PET) studies, an association between whole striatal dopamine synthesis and aberrant salience assessed by questionnaire seen in controls was absent in cannabis users (Bloomfield et al., 2016).

Aberrant salience has been postulated to underlie the development of psychotic-like symptoms in neuropsychiatric disorders (Kapur, 2003; van Os, 2009; Howes and Nour, 2016). Inappropriate allocation of salience or meaning to inconsequential stimuli, which is empirically associated with dysregulation of dopaminergic activity (Howes and Nour, 2016), has been suggested to provide a framework for understanding both the positive symptoms of schizophrenia and sub-clinical psychotic-like symptoms (Kapur, 2003). This model proposes that the development of psychotic symptoms is preceded by a prodromal period characterised by abnormal allocation of salience to otherwise neutral stimuli (Chun et al., 2019; Neumann et al., 2021).

The suggestion that inappropriate focus on irrelevant stimuli is central to psychosis is supported by studies of associative learning phenomena including latent inhibition (LI), both in psychometric studies of schizotypy in the general population and patient studies (LI; Baruch et al., 1988; Gal et al., 2009; Granger et al., 2012), Kamin Blocking (KB; Jones et al., 1992; Moran et al., 2003, Moran et al., 2008), and, to a lesser extent, overshadowing (Granger et al., 2012). In overshadowing (OS), two stimuli which differ in terms of salience (perceptual or informational) are presented as a compound stimulus and paired with an unconditioned stimulus (US). The OS effect refers to the observation that the more salient stimulus becomes a strong predictor of the US, likely because it captures more attention than the less salient element of the compound stimulus (Mackintosh, 1975). Studies conducted in animals have shown that OS is disrupted (i.e. increased learning to the less salient, overshadowed stimulus) by the indirect dopamine agonist amphetamine, which exerts psychotomimetic effects (O'Tuathaigh and Moran, 2002, O'Tuathaigh et al., 2003). While no studies to date have examined OS in people with a diagnosed psychotic disorder, OS score variation in university students has been associated with the scores on the ‘Unusual Experiences’ dimension of the Oxford-Liverpool Inventory of Feelings and Experiences (O-LIFE) schizotypy scale (Granger et al., 2012); this dimension is considered to relate to the positive symptoms of schizophrenia.

Some studies that have reported aberrant salience in schizophrenia have utilised self-report instruments such as the Aberrant Salience Inventory (ASI; Cicero et al., 2010). Others have employed behavioural, task-based measures of salience such as the Salience Attribution Test (SAT) (Roiser et al., 2009). The SAT task is a probabilistic learning task that measures abnormal salience as bias towards one of two cues that are of equal reward probability controlling for differences in simple reinforcement learning. Frequent cannabis use is associated with higher ASI scores (O'Tuathaigh et al., 2020) and others have reported a significant association between cannabis dependency/abuse status and high implicit aberrant salience scores in the SAT task (Bloomfield et al., 2016; Ricci et al., 2024). However, there is a lack of clarity around the extent to which various measures map onto overlapping constructs (Chun et al., 2019). Specifically, it has been suggested that the concept referred to as “aberrant salience” comprises dissociable processes (pre-attentive, attentional, affective), only some of which may be selectively disrupted by exposures to psychotogens (Chun et al., 2019; Wijayendran et al., 2018). Distinct SAT measures have been associated with variation in LI / learned irrelevance (LIrr) task performance, suggestive of common underlying processes (Schmidt and Roiser, 2009). However, while measures like LI, KB, and OS share associative/attentional mechanisms and a requirement to filter out irrelevant stimuli, salience allocation in OS is uniquely determined by perceptual effectiveness rather than the integration of information about prior stimulus pre-exposure reinforcement contingencies. As overshadowing does not depend upon prior reinforcement contingencies it represents a good test of “pure” abberant salience and might be expected to correlate with SAT performance. These underlying differences highlight the importance of empirical examination of the relationship between apparently overlapping salience constructs and how they may be differentially affected by risk factors, including history of cannabis use.

In the current study we examined associations between (a) cannabis use and OS performance; (b) salience measures across the OS task and the SAT task; (c) OS and self-report indices of aberrant salience and schizotypy. Based on previous reports on effects of long-term cannabis use in cue competition paradigms, it was hypothesised that heavy cannabis use would be associated with disrupted assignment of associative value to less salient stimuli in the OS task. Both the SAT and OS involve computation and tracking of reinforcement associations and responding appropriately (Katthagen et al., 2016). Therefore, it was also hypothesised that investigating the association between validated measures of salience attribution (SAT) and learning phenomena thought to tap salience processing will help to disentangle salience attribution effects from deficits in alternative cognitive domains. An additional aim was to examine the association between OS and both the ASI and schizotypy measures in a cross-sectional population.

## 2 Methods

In this cross-sectional analysis, participants were recruited using the Prolific (www.prolific.co) platform, a participant pool for online experiments with a participant verification system (Palan and Schitter, 2018). All participants were invited to participate in a multi-part study of the relationship between lifestyle factors and cognitive function. Firstly, participants completed a questionnaire battery to assess their schizotypy status, aberrant salience experiences and cannabis use history, as well as a range of demographic items (age, sex, nationality, education level). Participants then followed the link to complete the OS task. 1–2 days after completion of the control task (see description of OS task), participants were invited to complete the SAT task. The inclusion criteria for the study were (i) individuals aged between 18 and 55 years old and (ii) from a predominantly English-speaking location. Participants were excluded if they declared (i) a history of neurological disease or brain injury and/or (ii) having been formally diagnosed with a psychiatric illness. Ethical approval was obtained from the Social Research Ethics Committee of University College Cork, Ireland. All participants provided informed consent and were compensated £10 for their time.

### 2.1 Questionnaire measures

*Schizotypy:* Schizotypy status for each participant was assessed using the Schizotypal Personality Questionnaire (SPQ; Raine, 1991). This 72-item [i.e. “dichotomous” item (yes/no)] questionnaire identifies nine schizotypal traits: ideas of reference, odd beliefs/magical thinking, unusual perceptual experiences, suspiciousness/paranoid ideation, eccentric/odd behaviour and appearance, no close friends, social anxiety, odd speech, and constricted affect. These nine traits or sub-scales load onto three separate dimensions: Cognitive-perceptual/Positive, Interpersonal, and Disorganised (Raine, 1991; Raine et al., 1994).

*Aberrant salience:* The ASI is a 29-item self-report measure of aberrant salience experiences that generates a five-factor model: Increased Significance, Senses Sharpening, Impending Understanding, Heightened Emotionality, and Heightened Cognition (Cicero et al., 2010). These five factors also make up a single second-order factor, whereby a total ASI score may be calculated based on summation of scores from each factor.

*History of cannabis use* The Cannabis Experience Questionnaire modified version (CEQmv; Di Forti et al., 2009) was used to measure experiences, both past and present, of cannabis use. Relevant variables collected included age at first use, lifetime cannabis consumption, current cannabis consumption (defined as frequent use of cannabis consumption during the previous 12 months), frequency of use, and use of other substances.

Classification of cannabis use frequency was as described previously (Dawes et al., 2021, Dawes et al., 2022) whereby participants were presented with the following options: (a) lifetime use (“ever vs. never”), (b) current use (frequent use during previous year, yes/no), or (c) cannabis use frequency (5-level ordinal variable; every day, more than once a week, a few times each month, a few times each year, only once or twice ever).

### 2.2 Overshadowing

**Fig. 1:**
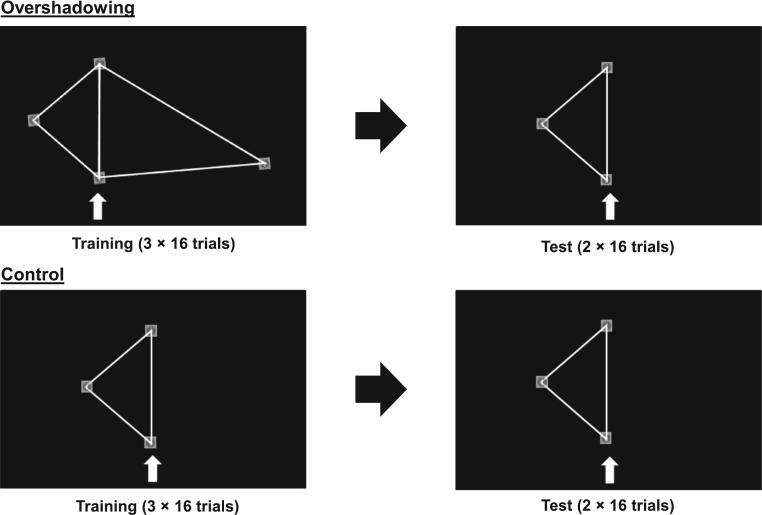
Description of the OS task.

OS was assessed using a within-subjects geometric learning task, as described previously (Prados, 2011; Granger et al., 2012). The task was developed and delivered using the cloud-based online experiment builder Labvanced (http://www.labvanced.com). The following two geometric shapes were employed: a scalene triangle (internal-angles: 85°, 60°, 35°); isosceles triangle (internal-angles: 50°, 50°, 80°). All other stimulus parameters were as presented previously (Granger et al., 2012). In this task, previous studies have shown that the scalene triangle is more salient than the isosceles triangle such that when it is used as the overshadowing cue it disrupts learning to the target (isosceles) triangle, i.e. it results in a robust OS effect (Prados, 2011; Granger et al., 2012). The task is delivered across five contiguous blocks of 16 trials (Fig. 1). In the first three ‘training’ blocks both triangles are presented as composite image and the participant is required to work out the ‘correct’ corner (chosen arbitrarily by the experiment), which remained stable throughout despite the composite image orientation shifting from trial to trial. They respond by using the mouse to click on the correct corner and a correct response is signalled by a round of applause. After the three training blocks have been completed, the ‘test’ blocks (2 × 16 trials) involve presentation of the less salient stimulus (the isosceles triangle) and the participant is required to select the correct corner. In the test trials, no auditory feedback is provided in relation to whether the correct response was selected. The control task follows the same procedure as the OS task except that the isosceles triangle is presented alone, i.e. it measures simple learning. All participants completed the control task 24–30 h after completing the OS task, as previous studies demonstrated that a more robust OS effect is observed where that task order is followed (Granger et al., 2012). In both task conditions (OS, control), percentage of correct responses for each trial block is recorded and presented. Participants were excluded from the analysis if they failed to achieve a criterion of 70 % correct responses in the third training block of trials.

(Adapted from Granger et al., 2012).

### 2.3 Salience Attribution Test

The SAT was conducted as described previously (Roiser et al., 2009) with minor adaptations for online delivery. These changes involved modification of on-screen instructions to ensure it could be completed independently. The task was developed and delivered using the cloud-based online experiment builder Pavlovia (http://www.pavlovia.org). In each SAT trial participants are exposed to a cue that precedes a probe, to which they need to respond in order to receive in-task reward (i.e. money). Participants are notified that availability of reward is dependent upon preceding cue characteristics and that the magnitude of the reward is based on the reaction time (RT) of their responses. There are 84 trials in total, divided across two blocks, and cues vary based on type (colours, animals/objects), where only colour predicts reward (87.5 % likelihood of reward for red vs 12.5 % for blue cues); in contrast, cue type is unrelated to reward contingencies as both types (animals and objects) are both reinforced at an equal level (50 %). After each block, explicit salience processing was measured by asking participants to estimate the reinforcement likelihood for each cue characteristic on a visual analogue scale (VAS; 0–100 %). Adaptive salience is measured by the difference in RT (implicit adaptive salience; ms) or VAS (explicit) between high-reward and low-reward cue trials (red vs blue trials). Aberrant salience is measured by the difference in RT (implicit) or VAS rating (explicit) for the equally irrelevant features (animals vs objects). Relevant and rewarded characteristics were balanced across participants.

### 2.4 Data analysis

Differences across OS performance stages and between cannabis groups (i.e., cannabis users and non-users) were assessed using mixed ANOVAs. Greenhouse-Geiser corrections were applied where necessary, but normality violations did not lead to non-parametric alternatives, as simulation studies suggest the robustness of ANOVAs against violations at suitable sample sizes (Schminder et al., 2010). Multiple regression analyses assessed whether SPQ and ASI scores predicted the magnitude of the overshadowing effect. For the SAT, Bayesian Spearman correlations were calculated between task metrics and SPQ and ASI subscale scores. Lastly, repeated-measures ANOVAs were used to compare differences in SAT Reaction Time (implicit salience) and Visual Analogue Scale scores (explicit salience) between blocks and cannabis use status.

## 3 Results

A total of 351 participants completed the overshadowing task, after which those who failed to achieve the pre-specified criterion of 70 % correct training responses were removed, assumed to be due to inattention or miscomprehension (*N* = 280). For the SAT, only a smaller group of participants completed the additional task in which all participants were included (*N* = 149). All participants had full data for both the SPQ and ASI survey. The mean age was 31.1 years (SD = 7.0, 46.1 % female). The prevalence of lifetime cannabis use was 62.5 % (*N* = 175), and 16.4 % (*N* = 46) met the definition of a current user. A full description of the socio-demographic and cannabis use history for all participants (N = 280) included in the final analysis is presented in Supplementary Table 1.

### 3.1 Overshadowing

#### 3.1.1 Task validation

**Fig. 2:**
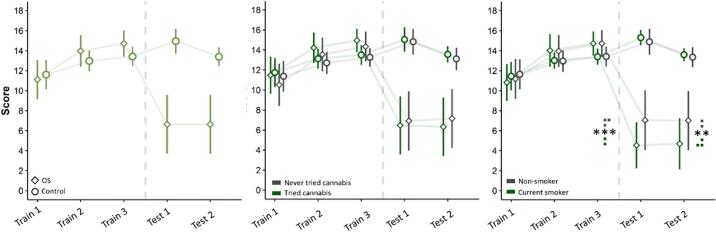
Performance in the OS task. Left: Scores for the OS stimulus were significantly lower than those for the control stimulus in the testing trial blocks. Centre: The same patterns of effects were found when dividing participants into non-smokers and individuals who had tried cannabis (i.e. lifetime cannabis use). Right: Current cannabis users had lower scores for the OS stimulus compared to non-cannabis users, but only during the testing trials. Values represent means and standard deviations; ** *p* < .01, *** *p* < .001.

To validate the OS effect, we compared responses between the control and OS conditions across the five blocks of 16 trials (three training and two testing) using a repeated-measure ANOVA with 280 participants. The analysis revealed a significant effect of condition (*p* < .001, η^2^p = 0.428), block (*p* < .001, η^2^p = 0.426) and a significant interaction between condition and block (*p* < .001, η^2^p = 0.550), all at large effect sizes. For the variable of condition, planned comparisons revealed that the OS condition (*M* = 10.6) had lower scores than the control condition (*M* = 13.3, *p*_*Holm*_ < 0.001, Cohen's *d* = 0.75). For block, each consecutive block was significantly different from the preceding. Specifically, scores increased from Training block 1 to Training block 2 (*p*_*Holm*_ < 0.001, *d* = 0.58) and again at Training block 3 (*p*_*Holm*_ <. 001, *d* = 0.17). The transition from Training block 3 to Test block 1 saw a significant decrease in scores at a large effect size (*p*_*Holm*_ <. 001, *d* = 0.92), which further decreased at Test block 2 (*p*_*Holm*_ < 0.001, *d* = 0.22). In terms of the condition * block interaction, the groups did not significantly differ at Training block 1 (*p*_*Holm*_ = 0.454, *d* = 0.13), but did at both Training block 2 (*p*_*Holm*_ = 0.007, *d* = 0.28) and Training block 3, (*p*_*Holm*_ < 0.001, *d* = 0.37), with the OS group having higher scores. During the testing phase, this relationship sharply switched, with the OS group having significantly lower scores at Test block 1 (*p*_*Holm*_ < 0.001, *d* = 2.34) and Test block 2 (*p*_*Holm*_ < 0.001, *d* = 1.90) at large effect sizes. Overall, scores were markedly different at the testing phases, indicating that OS was observed in the total sample (Fig. 2, left).

#### 3.1.2 Cannabis use and OS

Next, to assess the potential effect of cannabis use on OS, lifetime cannabis use or current cannabis use was added as an additional variable to the previous ANOVA model as a between-participants factor (2 × 2 × 5 ANOVA). In all following ANOVAs, the effects of trial and condition remained significant and thus the details are not repeated. The main effect of lifetime cannabis use was not significant (*p* = .223, η^2^p < 0.01), nor were the two-way interactions between lifetime cannabis use and condition (*p* = .75) and trial (*p* = .09) or the three-way interaction between all three variables (*p* = .13). This suggests that people who have never tried cannabis and those who have tried cannabis do not show differing OS effects (Fig. 2, middle).

The process was then repeated with current cannabis use as the between-subjects factor (Fig. 2, right). The main effect of current cannabis use was not significant (*p* = .13, η^2^p = 0.009). However, there was a significant three-way interaction between cannabis, block, and condition (*p* = .007, η^2^p = 0.02). Post-hoc *t*-tests indicated that cannabis users had lower scores for the OS stimulus at Test block1 (*p*_Holm_ = 0.001, *d* = 0.72) and Test block 2 (*p*_Holm_ = 0.003, *d* = 0.68). No significant differences were found at the three training blocks (*p* > .900, *d* < 0.07, nor in any of the blocks for the control condition (*p* > .9, *d* < 0.12). These relationships persisted even when co-varying the three SPQ scales and the five ASI scales in two further mixed-ANCOVAs, indicating this relationship to be independent of these measures. Together, this suggests that cannabis users show a stronger OS effect than non-users.

#### 3.1.3 Schizotypy and OS

To assess the influence of schizotypy on OS, two indices were calculated to simplify the analysis. This involved subtracting the third training block scores (i.e. Training block 3) from the final testing block scores (i.e. Test block 2) for both the control and OS conditions. A paired-sample Wilcoxon test revealed a significant difference between these two indices (*p* < .001, *r*_*rank-biserial*_ = 0.951), which also supported the presence of overshadowing. Multiple regressions were conducted to investigate whether the three schizotypy dimensions (Cognitive Perceptual, Interpersonal, and Disorganised) predicted the control and OS indices. For the control index, none of the three schizotypy scales significantly predicted scores (*p* > .707, β < 0.03) and Bayes Factors constituted strong evidence to accept the null hypothesis (BF_01_ > 15.6). Similarly, none of the nine SPQ subscales predicted the control index (*p* > .163, β < 0.15) and the Bayesian analysis constituted very strong evidence to accept the null hypothesis for all scales (BF_01_ > 31). This process was repeated for the OS index, which again indicated none of the three SPQ scales to significantly predict OS index scores (*p* > .554, β < 0.46), with strong evidence to accept the null hypothesis (BF_01_ > 12.3). In terms of subscales, no significant prediction was found (*p* > .350, β < 0.08), with very strong evidence to accept the null hypothesis (BF_01_ > 36).

#### 3.1.4 ASI and OS

To assess the influence of self-reported aberrant salience on overshadowing, the same process was repeated using the ASI subscales. For the control index, none of the five subscales significantly predicted scores (*p* > .149, β > −0.13) and there was strong evidence to accept the null hypothesis (BF_01_ > 14.5). For the OS index, the same null findings were replicated (*p* > .279, β < 0.124, BF_01_ > 20.8).

### 3.2 Salience Attribution Task (SAT)

#### 3.2.1 Associations with OS, schizotypy, and ASI

For the SAT analysis, a correlation matrix was created between SAT performance metrics and overshadowing, schizotypy, and ASI (*N* = 149). This analysis revealed that scores in the first OS training trial were significantly correlated with higher explicit aberrant salience scores in SAT Block 1 (*r*_*s*_ = 0.21, BF_10 =_ 4.31) and Block 2 (*r*_*s*_ = 0.32, BF_10_ = 601), as well as explicit adaptive salience scores in SAT Block 2 (*r*_*s*_ = 0.30, BF_10 =_ 163). However, the remaining comparisons largely supported the null hypothesis (BF_10_ < 0.3). Table 1 summarises these findings.

**Table 1:** Bayesian correlation matrix between SAT variables and overshadowing, schizotypy and aberrant salience.

	Block 1				Block 2			
Variable	Implicit adaptive	Implicit aberrant	Explicit adaptive	Explicit aberrant	Implicit adaptive	Implicit aberrant	Explicit adaptive	Explicit aberrant
OS training 1	0.06^N^	−0.04^N^	0.13^I^	0.20*	0.0^N^	−0.19^I^	**0.30**^**D**^	**0.32**^**D**^
OS training 2	0.15^I^	−0.02^N^	0.06^N^	0.08^I^	−0.02^N^	−0.05^N^	0.12^I^	0.12^I^
OS training 3	0.02^N^	−0.13^I^	0.09^I^	0.06^N^	0.04^N^	0.13^I^	0.11^I^	0.12^I^
OS test 1	−0.09^I^	−0.16^I^	0.06^N^	0.07^N^	−0.05^N^	−0.17^I^	0.12^I^	0.11^I^
OS test 2	−0.10^I^	−0.14^I^	0.05^N^	0.08^I^	0.04^N^	−0.18^I^	0.14^I^	0.10^I^
Cognitive Perceptual	−0.10^I^	0.07^N^	−0.01^I^	−0.11^I^	0.04^N^	0.16^I^	−0.06^N^	−0.07^N^
Interpersonal	−0.03^N^	−0.10^I^	0.07^N^	0.08^I^	0.02^N^	0.08^I^	0.10^I^	0.10^I^
Disorganised	0.02^N^	0.08^I^	0.02^N^	0.03^N^	−0.02^N^	0.07^N^	0.02^N^	0.02^N^
Increased significance	−0.04^N^	0.06^N^	−0.12^I^	−0.10^I^	−0.05^N^	0.09^I^	0.01^N^	0.02^N^
Senses Sharpening	0.05^N^	−0.15^I^	−0.14^I^	−0.05^N^	0.14^I^	−0.07^I^	0.03^N^	−0.05^N^
Impending Understanding	0.09^I^	0.02^N^	−0.14^I^	−0.12^I^	0.02^N^	0.01^N^	0.06^N^	0.01^N^
Heightened Emotionality	−0.05^N^	−0.07^N^	−0.04^N^	0.01^N^	−0.01^N^	0.11^I^	−0.09^I^	−0.06^I^
Heightened Cognition	0.01^N^	0.01^N^	−0.06^N^	−0.11^I^	0.12^I^	0.014^I^	−0.07^I^	−0.08^I^

Note: N = BF < 0.3, I = BF between 0.3 and 3, * = BF > 3, ** = BF > 10, *** = BF > 30, D = BF > 100.

#### 3.2.2 Implicit salience

**Fig. 3:**
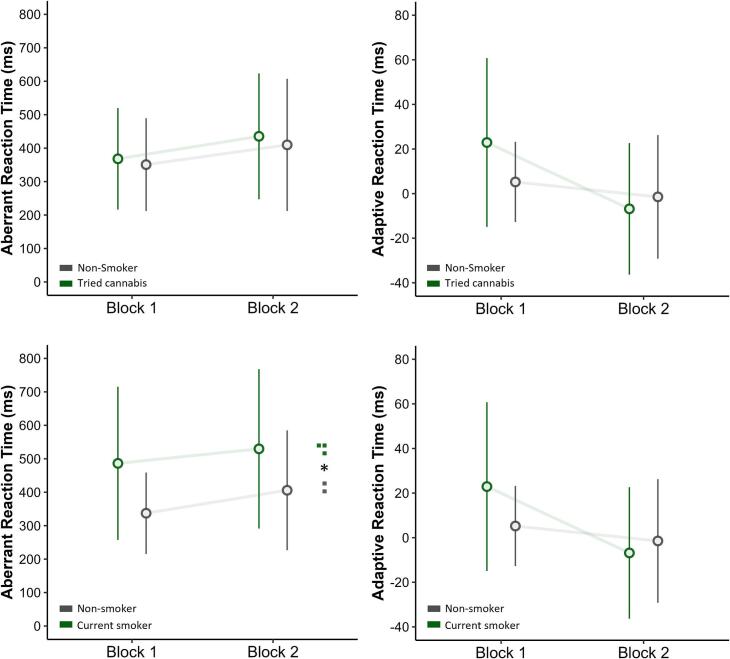
Plotted reaction times in the Salience Attribution Task (SAT). The figures are divided into lifetime cannabis use (top) and current cannabis use (bottom) and assess Aberrant RT (left) and Adaptive RT (right). Values represent means and standard deviations. Note: † = *p* < .1, * *= p* < .05.

To assess the potential effect of cannabis use on SAT performance, both the implicit (RT) and the explicit (VAS) outcomes were analysed separately using 2 × 2 repeated-measure ANOVAs with the first factor being cannabis use (Yes or No) and the second factor being SAT block (Block 1 or Block 2). For succinctness, only the effects related to cannabis use are outlined in detail. For lifetime cannabis use and implicit aberrant salience, the effects of lifetime cannabis use (*p* = .655) and the interaction with Block were non-significant (*p* = .904). The same pattern of results was seen for implicit adaptive salience, with neither the effect of lifetime cannabis use (*p* = .422) nor the interaction with Block being significant (*p* = .327). The process was repeated with current cannabis use as the between-subjects factor. For implicit aberrant salience, the effect of current cannabis user was significant (*p* = .024), with an increase in implicit aberrant salience reaction time (M = 463 ms) relative to non-users (M = 326 ms, *d* = 0.404). The interaction with Block was non-significant (*p* = .772). For implicit adaptive salience, neither the effect of current cannabis use (*p* = .344) nor the interaction between current cannabis use and Block was significant (*p* = .118). Fig. 3 summarises these relationships.

#### 3.2.3 Explicit salience

**Fig. 4:**
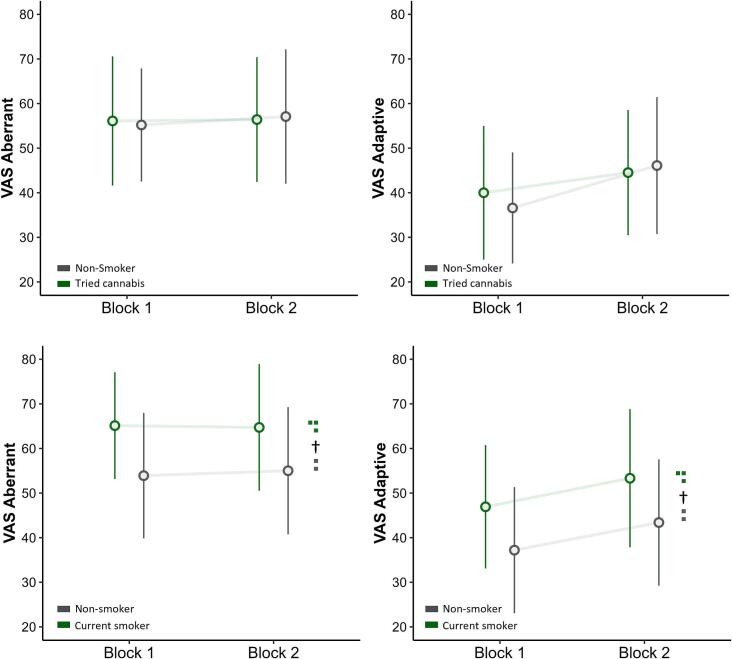
Plotted task VAS scores of the Salience Attribution Task (SAT). The figures are divided into lifetime cannabis use (top) and current cannabis use (bottom) and assess Aberrant RT (left) and Adaptive RT (right). Values represent means and standard deviations. Note: † = *p* < .1.

The above analyses were repeated with VAS score as the outcome variable. For explicit aberrant salience, neither the effect of lifetime cannabis use (*p* = .980) nor the interaction with Block was significant (*p* = .690). For explicit adaptive salience, the effect of cannabis (*p* = .836) and its interaction with Block were again non-significant (*p* = .253). The process was repeated for current cannabis use. For explicit aberrant salience, the effect of current cannabis use returned as trend (*p* = .064) and the interaction with Block interaction was non-significant (*p* = .763). For explicit adaptive salience, the effect of cannabis also returned as trend (*p* = .081) and its interaction with Block was also non-significant (*p* = .970). Fig. 4 summarises these relationships.

## 4 Discussion

This study revealed that online adaptation of the OS task was successful, in that participants' scores increased through the training blocks and that scores for the OS stimuli were significantly lower in the testing block. In terms of cannabis use history, while lifetime cannabis use did not affect OS task performance, those who currently use cannabis had significantly higher OS scores during the testing phase relative to those who do not use cannabis, at medium effect sizes. Levels of schizotypy traits or aberrant salience (i.e. ASI) scores did not mediate this relationship. No other significant differences were found between cannabis users and non-users.

For the SAT task, scores in the first training phase of the OS task significantly predicted higher explicit aberrant salience scores in SAT Block 1 and Block 2, as well as explicit adaptive salience scores in Block 2. While lifetime cannabis use (‘ever used’) did not affect implicit or explicit aberrant or adaptive salience values, current cannabis users showed a significant increase in implicit aberrant stimuli but no change in implicit adaptive salience relative to non-users. Current cannabis use did not significantly affect explicit aberrant or adaptive salience scores.

These results with the OS task are consistent with the hypothesis that cannabis users show abnormalities in cue competition paradigms that measure selective allocation of associative salience. It is also in agreement with reports that changes in aspects of associative learning performance are associated with longer and higher frequency of cannabis use (Solowij et al., 2002; Messinis et al., 2006; Becker et al., 2010). However, the direction of these effects of cannabis is contrary to expectations based on the schizophrenia-like over-attribution of motivational value to irrelevant environmental events. In previous studies with KB (Dawes et al., 2021) and LI (Dawes et al., 2022), neither lifetime nor current cannabis use was associated with changes across any of the performance measures. Both KB and LI involve a comparison of previous learning with current contingencies. In these cases, where distinct performance decrement profiles in these tasks are found in schizotypy (Granger et al., 2012; Moran et al., 2003), as well as in people with schizophrenia (Jones et al., 1992; Gal et al., 2009), the observed changes have been linked with impaired integration of past learning with current perception (i.e. the ‘comparator’ model; Gray, 1998). In contrast, in the OS task, participants are not forming associations with cues based on previous cues, but based on evaluation of the relative predictive values of stimuli of unequal perceptual salience. It has been suggested that OS and other cue competition task variants may involve higher order reasoning or contingency judgement processes (De Houwer et al., 2005). In the current study, it is notable that compound training trial performance in the OS task was significantly associated with selected explicit (and not implicit) aberrant and adaptive salience measures in the SAT task. The adaptive salience index measures judgement of the relative reward contingency values of the relevant cue characteristics. Only compound training performance was associated with only conscious appraisal of probabilistic stimulus-reinforcement associations in the SAT. This suggests that salience attribution in the OS task (and other variants, see Pickett et al., 2017) may involve some contribution of conscious, propositional knowledge. The possibility that the observed increase in OS in frequent cannabis users may reflect a downstream effect on a higher-order contribution of predictive salience to co-occurring stimuli merits further investigation.

Studies using the SAT in schizophrenia have reported deficits in adaptive salience relative to controls (Roiser et al., 2009; Abboud et al., 2016; Katthagen et al., 2016; Smieskova et al., 2015; Pankow et al., 2015). Although some reports have shown disrupted aberrant salience in schizophrenia (Katthagen et al., 2016; Pankow et al., 2015; Neumann et al., 2021), others results are less consistent (Roiser et al., 2009; Smieskova et al., 2015; Abboud et al., 2016); some have observed disruption of implicit but not explicit aberrant salience (Pankow et al., 2015), while others have observed the opposite effect (Roiser et al., 2009). In a comparison of SAT performance in 17 cannabis users and 17 controls, Bloomfield et al. (2016) reported no group differences were observed across any of the SAT measures, but within cannabis users they reported a significant positive relationship between cannabis-induced psychotic symptom severity and explicit aberrant salience scores, as well as a significant association between cannabis dependency/abuse status and high implicit aberrant salience scores. The current study included a larger sample but, in contrast with that of Bloomfield et al. (2016), cannabis use status was based solely on self-report data. Here, current cannabis users demonstrated an increase in implicit aberrant salience, but the sample size and self-report data precluded any subgroup analysis based on dependency status. It has been suggested that implicit aberrant salience in the SAT reflects a stable bias towards an irrelevant cue feature (Katthagen et al., 2016); this bias may represent a secondary effect to a more disorganised aberrant salience that is reflected in the participant sticking to one available strategy in a complex situation.

In relation to earlier comparisons regarding cannabis effects (or lack thereof) on LI and KB (Dawes et al., 2021, Dawes et al., 2022), the present findings regarding current cannabis effects on aberrant salience further supports doubt as to the degree of conceptual overlap between the ‘comparator’ model and the aberrant salience construct (Schmidt and Roiser, 2009). Specifically, Schmidt and Roiser (2009) compared performance in the SAT and a learned irrelevance task that measures the ability to disregard stimuli previously presented without consequence. They demonstrated that while learned irrelevance and adaptive (but not aberrant) salience appear to be associated, the former was dissociable from aberrant salience as measured in the SAT.

As reported previously for LI (Chun et al., 2019; Dawes et al., 2022), none of the OS measures were associated with self-report measures of aberrant salience or positive schizotypy; furthermore, scores across either scale did not moderate the reported effect of cannabis use on OS performance. These data suggest that the consistently observed effects of cannabis use on self-reported aberrant salience or schizotypy scale scores (e.g. O'Tuathaigh et al., 2020; Dawes et al., 2021) are distinct from effects on OS task performance. These results contrast with previously observed negative association between the “Unusual Experiences” O-LIFE schizotypy dimension (Granger et al., 2012) and OS using the same task version applied in the current study. However, no association was reported between any of the O-LIFE schizotypy dimensions and OS performance using a different OS task (Pickett et al., 2017), supporting the contention that the relationship between OS and schizotypy is not as clear and prominent as it may be for other related learning phenomena such as LI and blocking (Pickett et al., 2017). Difference between the current study and earlier reports, such as Granger et al. (2012), may also relate to the sample population, where the current study sample encompasses more diverse socio-demographic and educational characteristics, or it may relate to use of different schizotypy scales (O-LIFE vs SPQ). Others have noted that the O-LIFE is rooted in the personality psychology tradition of schizotypy research, whereas the SPQ was created based on diagnostic criteria for schizotypal personality disorder (Polner et al., 2021).

Our findings agree with previous reports of a lack of association between SAT measures and ASI (Neumann and Linscott, 2018). Neumann et al. (2021) also reported no significant correlation between ASI and SAT in a sample of schizophrenia, anxiety, and control participants. Similarly, they reported no relationships between ASI or SAT and alternative measures of reinforcer sensitivity or motivational salience as measured in in the Effort Expenditure for Reward Task. Others have questioned the ability of the ASI as a scale to discriminate patients with psychotic symptoms from patients without such symptoms (Ballerini et al., 2022). The data support the proposition that there is “variance in construct definition” among measures of aberrant salience (Katthagen et al., 2016) and that caution should be applied when synthesising results across studies using currently available measures. Taken together, the present findings indicate an association between regular cannabis use and abnormalities in cue competition associative learning effects in a healthy adult sample.

## CRediT authorship contribution statement

**Christopher Dawes:** Conceptualization, Formal analysis, Investigation, Methodology, Writing – original draft, Writing – review & editing. **Samuel Joy McGreal:** Investigation, Methodology, Project administration, Writing – review & editing. **Shivika Marwaha:** Investigation, Methodology, Project administration, Writing – review & editing. **Jose Prados:** Methodology, Resources, Writing – original draft, Writing – review & editing. **Antoine Reheis:** Methodology, Project administration, Resources, Software, Writing – review & editing. **Alin Dumitrescu:** Investigation, Methodology, Project administration, Writing – original draft, Writing – review & editing. **John L. Waddington:** Conceptualization, Formal analysis, Investigation, Methodology, Writing – original draft, Writing – review & editing. **Paula M. Moran:** Conceptualization, Formal analysis, Investigation, Methodology, Project administration, Visualization, Writing – original draft, Writing – review & editing. **Colm O'Tuathaigh:** Conceptualization, Data curation, Formal analysis, Investigation, Methodology, Project administration, Resources, Validation, Visualization, Writing – original draft, Writing – review & editing.

## Declaration of competing interest

None.

## Appendix A Supplementary data

Supplementary material Image 1
