# Supplementary material for: Overshadowing and salience attribution in relation to cannabis use
Source: Schizophr Res Cogn. 2024 May 10;37:100315. doi: 10.1016/j.scog.2024.100315 (PMC11101976; doi:10.1016/j.scog.2024.100315)
Supplement: Supplementary file 1 — Supplementary material [file mmc1.docx]

**Supplementary Material**

**Supplementary Table 1** Characteristics of the sample population (*n=*280)

| ***Characteristic*** | *n* | ***%*** |
| --- | --- | --- |
| **Sex** |  |  |
| Female | 129 | (46.1%) |
| Male  Other | 147  4 | (52.5%)  (1.4%) |
| **Nationality** |  |  |
| Irish/British | 193 | (68.9%) |
| Other Europe | 31 | (11.1%) |
| North American | 34 | (12.1%) |
| Other/Not specified | 22 | (7.9%) |
| **Highest level of education** |  |  |
| Secondary level | 42 | (15.0%) |
| Post-secondary level | 44 | (15.7%) |
| Primary degree | 127 | (45.4%) |
| Masters/Doctoral degree | 67 | (23.9%) |
| **Family history of mental illness** |  |  |
| Yes | 80 | (28.6%) |
| No | 199 | (71.1%) |
| Not specified | 1 | (0.4%) |
| **Lifetime cannabis use** |  |  |
| Yes | 175 | (62.5%) |
| No | 105 | (37.5%) |
| **Current cannabis use** |  |  |
| Yes | 46 | (16.4%) |
| No | 234 | (83.6%) |
| **Age at first cannabis use** |  |  |
| Mean age (SD) | 17.6 | (3.2) |
| Range | 10-29 |  |
| **Frequency of cannabis use** |  |  |
| Every day | 16 | (5.7%) |
| Greater than once a week | 28 | (15.7%) |
| A few times each month | 28 | (25.7%) |
| A few times each year | 42 | (40.7%) |
| Only once or twice | 73 | (13.6%) |
| Never | 93 | (33.2%) |
|  |  |  |

*Figures presented are number (%) unless stated otherwise.*

**Supplementary Data 1**

**OS Task Validation**

For the variable of condition, planned comparisons revealed that the OS condition (*M* = 10.6) had lower scores than the control condition (*M* = 13.3, *p_Holm_* < .001, Cohen’s *d* = 0.75). For trial blocks, each consecutive trial block was significantly different from the preceding trial. Specifically, scores increased from Training trial 1 to Training trial 2 (*p_Holm_* < .001, *d* = 0.58) and again at Training trial 3 (*p_Holm_* <. 001, *d* = 0.17). The transition from Training trial 3 to Test trial 1 saw a significant decrease in scores at a large effect size (*p_Holm_* <. 001, *d* = 0.92), which further decreased at Test trial 2 (*p_Holm_* < .001, *d* = 0.22). In terms of the condition * trial interaction, the condition did not significantly differ at Training trial 1 (*p_Holm_* = .454, *d* = 0.13), but did at both Training trial 2 (*p_Holm_* = .007, *d* = 0.28) and Training trial 3, (*p_Holm_* < .001, *d* = 0.37), with the OS condition having higher scores. During the testing phase, this relationship sharply switched, with the OS condition having significantly lower scores at Test trial 1 (*p_Holm_* < .001, *d* = 2.34) and Test trial 2 (*p_Holm_* < .001, *d* = 1.90) at large effect sizes.
